# Characterization of Volcanic Cloud Components Using Machine Learning Techniques and SEVIRI Infrared Images

**DOI:** 10.3390/s22207712

**Published:** 2022-10-11

**Authors:** Federica Torrisi, Eleonora Amato, Claudia Corradino, Salvatore Mangiagli, Ciro Del Negro

**Affiliations:** 1 Istituto Nazionale di Geofisica e Vulcanologia, Sezione di Catania, Osservatorio Etneo, 95125 Catania, Italy; 2 Department of Electrical, Electronic and Computer Engineering, University of Catania, 95125 Catania, Italy; 3 Department of Mathematics and Computer Science, University of Palermo, 90123 Palermo, Italy

**Keywords:** volcano remote sensing, machine learning classifier, volcanic cloud, volcanic ash, SO_2_ gas, geostationary satellite, support vector machine

## Abstract

Volcanic explosive eruptions inject several different types of particles and gasses into the atmosphere, giving rise to the formation and propagation of volcanic clouds. These can pose a serious threat to the health of people living near an active volcano and cause damage to air traffic. Many efforts have been devoted to monitor and characterize volcanic clouds. Satellite infrared (IR) sensors have been shown to be well suitable for volcanic cloud monitoring tasks. Here, a machine learning (ML) approach was developed in Google Earth Engine (GEE) to detect a volcanic cloud and to classify its main components using satellite infrared images. We implemented a supervised support vector machine (SVM) algorithm to segment a combination of thermal infrared (TIR) bands acquired by the geostationary MSG-SEVIRI (Meteosat Second Generation—Spinning Enhanced Visible and Infrared Imager). This ML algorithm was applied to some of the paroxysmal explosive events that occurred at Mt. Etna between 2020 and 2022. We found that the ML approach using a combination of TIR bands from the geostationary satellite is very efficient, achieving an accuracy of 0.86, being able to properly detect, track and map automatically volcanic ash clouds in near real-time.

## 1. Introduction

Any emission of gasses and particles from a volcano that reach the atmosphere is referred to as a volcanic cloud. The principal component of volcanic clouds is the ash derived from magmatic material that emerges as solid into large particles [1], while the major volcanic gasses are water vapor (H_2_O), sulfur dioxide (SO_2_) and carbon dioxide (CO_2_). H_2_O and CO_2_ are widely distributed in the atmosphere, making it difficult to distinguish these gasses of volcanic origin from background quantities. Therefore, the approaches proposed to characterize the major components of a volcanic cloud are mainly focused on the detection and quantification of ash and SO_2_ [2]. The focus on ash and SO_2_ is mainly driven by the impacts that these components have on the atmosphere and the environment [3,4]. Moreover, both of these emissions can be hazardous to public health and aviation activities [5,6,7]. SO_2_ is converted to sulfuric acid, forming small droplets that affect the Earth’s radiation balance by reflecting solar radiation away from the surface [8]. The resulting disturbance to the Earth’s radiation balance affects surface temperatures through direct radiative effects as well as through indirect effects on atmospheric circulation, resulting in important natural causes of climate change on many timescales [9]. Volcanic ash can also affect the radiation balance, but since it only lasts a few days in the atmosphere, its effects are mostly local. An eruptive column still carrying these hot particles can produce a pyroclastic flow, which can be deadly to those unlucky enough to be at the base of the volcano [10,11,12]. Nevertheless, volcanic ash is a hazard for aircraft, because it can damage the jet engines [13].

If inhaled, volcanic ash can damage the lungs and cause breathing problems. Moreover, ash particles can scratch the surface of eyes and skin. SO_2_ gas can irritate the skin, tissues, eyes, nose and throat, sometimes producing respiratory distress [14]. It is well known that the dispersion of these components also impacts human health, not only in the area surrounding a volcano, since volcanic ash is frequently dispersed even thousands of kilometers away from the source [15].

Currently, satellite observations are widely exploited to monitor volcanic clouds [16,17,18]. The most useful sensors for volcanic ash and gas detection span the infrared (IR) spectral regions [19]. In particular, the IR region between 8–12 µm is the most proper for this purpose since SO_2_ gas is absorbed in the band around 8.6 µm, while ash particles exhibit dispersive behavior in the same range [20]. These sensors operate on geostationary and polar satellites. Polar satellites are sun-synchronous, relatively close to the Earth and provide a high spatial resolution (but low temporal resolution). On the contrary, geostationary satellites are geosynchronous, monitor the same area of the Earth and have a high temporal resolution (but low spatial resolution). Both satellites are useful for volcanic ash and SO_2_ remote sensing, but the high temporal resolution of geostationary observations allows one to continuously monitor, and in near real time, volcanic activity and therefore to track the evolution of a volcanic cloud [21]. For these reasons, a good candidate for the purpose of detecting and characterizing a volcanic cloud is the Spinning Enhanced Visible and Infrared Imager (SEVIRI) sensor on board the Meteosat Second Generation (MSG) geostationary satellites. SEVIRI has a good spectral resolution in the infrared region and a high-temporal resolution, which allows for an almost continuous 24 h monitoring.

The first study for the detection of volcanic ash clouds using satellite data was conducted by Sawada [22]. In 1989, Prata [23,24] proposed the first operationally viable volcanic ash algorithm, called the reverse absorption (RA) method, which involves taking the brightness temperature difference (BTD) between the 11 and 12 µm IR channels. This method is also called the “split window” method, because the two IR channels selected are situated in the atmospheric window regions, where the effects of other atmospheric gases is small. The positive values of the BTD are related to the presence of meteorological clouds, while negative values for the presence of volcanic ash. When a volcanic ash cloud is present, the brightness temperature (BT) measured above the ash cloud increases with increasing wavelength through the window, while the reverse is true for a layer of ice or water particles (meteorological clouds) [25]. Therefore, the wavelength dependence of the absorption by ash is the opposite with respect to water and ice [26]. The advantage of the RA method is that, since it exploits IR bands, it is applicable both day and night. This approach fares poorly in several circumstances, because the BTD values depend on the height of the eruption cloud, the mass per unit area of fine ash within the cloud and the size of the ash particles [27,28]. For this reason, the method for the detection of a volcanic ash cloud was improved with the three-band IR algorithms, which include channels centered near 4 µm [29,30] and 13.3 µm [31].

More robust methods to detect volcanic ash were developed successfully, using data from the IR channels of geosynchronous satellite sensors such as SEVIRI [32] and GOES [33]. Prata and Kerkmann [34] performed the first simultaneous measurements of volcanic ash and SO_2_ using five infrared channels of SEVIRI centered at 6.2, 7.3, 8.5, 11 and 12 µm. Moreover, SEVIRI IR bands were also exploited to perform a quantitative characterization of the volcanic cloud components and to reconstruct the ash and SO_2_ flux emitted during some recent eruptions at Mt. Etna (Sicily, Italy) volcano [35,36,37]. However, these approaches exploit a microphysical model of an ash cloud and parameters determined from Modtran 3 [38] simulations. One of the key parameters is the plume height, retrieved comparing the BT of the 11 μm band (BT_11_) of the opaquest pixels with the temperature profile. However, if the volcanic cloud starts to dilute and the pixels become partially transparent, this procedure fails, underestimating the cloud height [39]. Therefore, these approaches need parameters that cannot always be calculated automatically.

The discrimination between volcanic and weather clouds is a major issue, because in the region where the two types of clouds overlap, the BTD can be negative both for volcanic and for meteorological clouds. For this reason, correction procedures were applied [40,41,42].

Currently, new advanced approaches to set the best BTD threshold for the detection of volcanic ash clouds were developed, such as machine learning (ML) techniques. ML algorithms offer an innovative paradigm to automatically process a huge amount of satellite data for volcanology applications [43,44,45]. In this way, it is possible to create the volcanic cloud mask automatically, avoiding the manual time-consuming drawing. ML techniques can be used for classification purposes since they are able to learn complex patterns and trends from data. The pixel classification of volcanic cloud components can be a useful procedure because from the processing of satellite images acquired with high temporal resolution it is possible to follow the evolution of a volcanic cloud, its composition and how its components are dispersed in the atmosphere. The extraction of information related to a volcanic cloud using satellite data is a challenging procedure, because sometimes it is difficult to distinguish volcanic ash clouds from more common meteorological clouds, such as thin cirrus clouds, and in most cases, it is necessary the supervision of an operator to discriminate the components of a volcanic cloud. Supervised ML approaches learn from a training dataset, which includes a set of coupled inputs and expected outputs. Furthermore, an unsupervised ML algorithm does not use a training dataset but extracts common features from the input data based on their similarity.

We recently explored the potential of ML techniques to detect volcanic clouds by using both supervised and unsupervised approaches, namely support vector machine (SVM) and K-means [46,47]. As expected, the results show that SVM outperforms K-means since the intervention of the human to choose and label the training samples makes the model much more accurate and moreover generalizable.

Here, we take a step forward toward the full characterization of the volcanic clouds. We propose a SVM-based method to both detect a volcanic cloud and to discriminate its components, namely, to classify its pixels as rich of ash, rich of SO_2_ or characterized by mixed components (ash, SO_2_ and other). The SVM algorithm was implemented in the Google Earth Engine (GEE) platform [48]. This cloud computing platform offers unique opportunities for remote sensing data collection, processing, analysis, and visualizations at a regional scale with direct access to a multi-petabyte analysis-ready data catalogue. The proposed SVM exploits as input a combination of bands in the infrared regions of images acquired by the sensor SEVIRI and returns as output an image with four classes: ash, SO_2_, mix of ash and SO_2_ (or simply mix), and background. This ML algorithm was applied to some of the paroxysmal explosive events that occurred at Mt. Etna between 2020 and 2022, considering a separate dataset as training and as testing.

## 2. Materials

### 2.1. 2020–2022 Etna Paroxysmal Events

**Figure 1:**
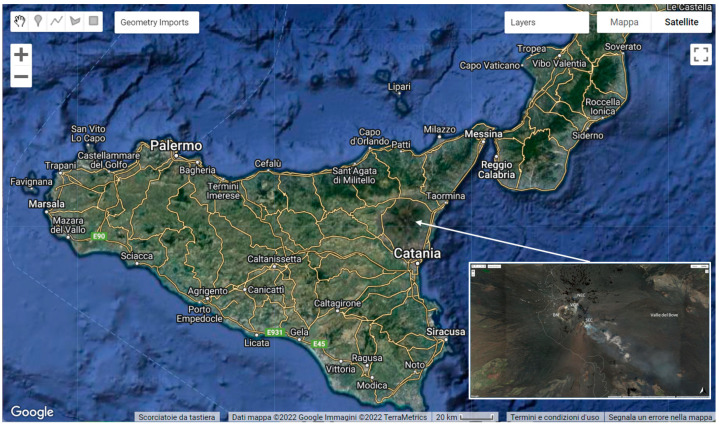
General Mt. Etna volcano in Sicily and relative summit area (white box), with four summit craters, Northeast Crater (NEC), Voragine (VOR), Bocca Nuova (BN) and Southeast Crater (SEC), and the Valle del Bove.

Mt. Etna (Figure 1) is the largest active volcano in Europe, and its activity often originates from its summit areas, which include four craters, named Northeast Crater (NEC), Voragine (VOR), Bocca Nuova (BN) and Southeast Crater (SEC), the most active crater since 1998 [49]. The youngest cone is the New Southeast Crater (NSEC), which began to grow after 2011 over the east flank of the SEC cone [50,51]. Eruptive activity after 2013 has led to the merging of the NSEC with the SEC [52]. Between 13 December 2020 and 21 February 2022, a new explosive phase took place at Mt. Etna, giving rise to 66 paroxysmal lava fountain episodes (from INGV weekly bulletins at www.ct.ingv.it, accessed on 1 July 2022) [53,54,55]. These events were characterized by Strombolian explosions, lava fountains, formation of short-lived lava flows and generation of eruptive columns [56]. An eruptive column is a cloud composed of ash, tephra and gasses emitted during a volcanic eruption. It can rise several kilometers above the vent of the volcano and can feed ash plumes. Ash plumes generated during the 2020–2022 Etna paroxysmal event reached an altitude of about 9–10 km above sea level and were spread by the wind into the atmosphere. The explosive events considered in this study are those characterized by an intense and violent activity that produced large and high volcanic clouds. These were on 23 February 2021, 12 March 2021, 9 August 2021 and 10 February 2022.

### 2.2. Satellite Data Sources

SEVIRI is an instrument onboard the Meteosat Second Generation (MSG) geostationary satellite, operated by EUMETSAT [57]. It measures radiances in 12 spectral channels, which cover the range of visible to the infrared with a spatial resolution of 3 km at the equator to 4.5 km at Mediterranean latitudes. The main advantages of SEVIRI is its high temporal resolution of 15 min for the full disc and 5 min in rapid scan mode, which mainly covers Europe [58]. For the detection and characterization of volcanic cloud components, only the thermal infrared (TIR) channels were considered, and in particular, the spectral bands centered at 8.7, 10.8 and 12.0 µm.

## 3. Methods

The SVM is a general supervised learning method used for classification and regression. The foundations of SVM have been developed by Vapnik [59] and gained popularity due to many promising features such as better empirical performance. The SVM algorithm aims to find the plane or set of hyperplane maximizing the distance between samples belonging to different classes closest to the boundary [60,61]. The SVM was already exploited by the authors to detect a volcanic cloud and has been shown to be more efficient than unsupervised algorithms such as K-means [47]. One alternative to the traditional SVM can be the Neural Networks (NN), which sometimes make classification problems much easier and faster to compute. NN have already been employed to detect volcanic clouds [62,63,64,65,66,67]. However, NN is not always the answer to all classification problems. SVM requires 2 parameters (*c* and γ, which will be described in Section 3.2), a kernel to map the input data to a higher dimensional space and few input data, which need minimal processing. Furthermore, NN requires several parameters that depend on the number of layers, a non-linear activation function and many input data, which generally need a lot of processing. Therefore, since we have limited data and a simple classification problem, we prefer to implement a SVM algorithm compared to other more complex algorithms such as NN because it produces results with easy implementations and in a quick way.

The objective of this work is to train a SVM model for a multi-class classification problem, in order to segment a combination of SEVIRI TIR bands, called Ash RGB (Red, Green, Blue) into 4 classes: pure ash cloud, pure SO_2_ cloud, mix cloud and background. In this way, it is possible to detect and characterize a volcanic cloud produced during a volcanic eruption. Once the model has been trained on some labeled samples and tested on new images, due to its high accuracy, it can be applied to any new images, without being trained again. The possibility of applying the model to new images makes it generalizable and applicable during a volcanic event even in near real time to monitor the evolution of a volcanic cloud.

We implemented the SVM model in GEE to automatically process and analyze SEVIRI Ash RGB images. The Ash RGB uses only infrared window channels, and therefore, it can be used both day and night for the detection and monitoring of volcanic ash as well as for sulfur dioxide gas.

**Figure 2:**
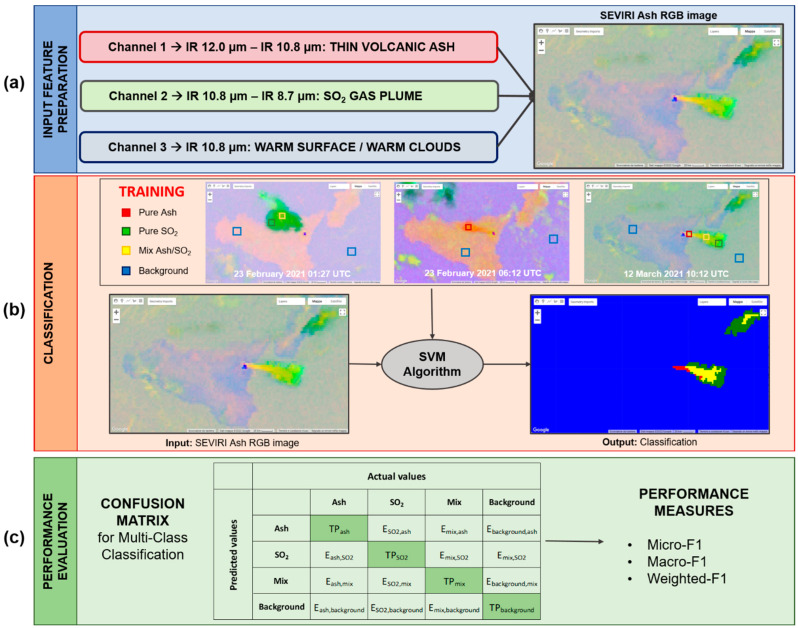
General scheme representing the three main steps of the support vector machine (SVM) algorithm written in Google Earth Engine (GEE): (**a**) Input Features Preparation, (**b**) Classification and (**c**) Performance Evaluation. The first step is to prepare the input feature of the SVM, obtained by opportunely combining the thermal infrared (TIR) bands centered at 8.7, 10.8 and 12.0 µm. The second is the implementation in GEE of the SVM algorithm to detect a volcanic cloud and identify its main components, while the last is the evaluation of the performance of the model using a confusion matrix.

In Figure 2, a general scheme of the ML algorithm proposed is reported. It represents the three main steps of our procedure: (a) the input feature preparation, i.e., the combination of the SEVIRI TIR band to construct the Ash RGB images, (b) classification, i.e., the design of the SVM classifier, and finally, (c) performance evaluation, to determine whether our model is working well or not.

### 3.1. Input Feature Preparation

The first step of our procedure is to prepare the input feature of the SVM algorithm. This step is fundamental since a discriminative set of features can properly separate the thin volcanic ash from cloud objects, provide SO_2_ detection, and allow for analysis of scenes containing a mix of ash and sulfur dioxide (also called mixed region).

SEVIRI images are provided by the EUMETSAT Data Store. In particular, we used the product MSG Level 1.5 Image Data, which corresponds to image data that have been corrected for all unwanted radiometric and geometric effects, has been geolocated using a standardized projection, and has been calibrated and radiance-linearized [68]. The Level 1.5 image, provided in a geostationary projection (GEOS Projection), were georeferenced to the reference system EPSG:4326—WGS 84.

By opportunely combining TIR bands centered at 8.7, 10.8 and 12.0 µm, it is possible to obtain an Ash RGB image highlighting the presence of the main components of a volcanic cloud. In particular, Ash RGB images are realized combining the brightness temperature (BT) of the three SEVIRI TIR channels in this way: red: BT_12.0_ − BT_10.8_; green: BT_10.8_ − BT_8.7_; blue: BT_10.8_ [69]. The channel combination in the red beam is the reverse of the “split window” method [23]: thin volcanic ash tends to have a strong reddish color, while meteorological clouds have no contribution. The green channel emphasizes the presence of SO_2_ (marked by green pixels), since it compares the SO_2_ absorption band at 8.7 µm with the non-absorbing 10.8 µm band. Finally, the 10.8 µm in the blue beam provides a high contrast background for ash detection and removes the influence of cumulonimbus clouds. Therefore, depending on the concentration, red pixels indicate the presence of thin volcanic ash, green pixels the presence of SO_2_, while yellow pixels mark the mixed regions of a volcanic cloud characterized by both ash and SO_2_. There are some limitations related to an Ash RGB image, such as the difficult identification of ash and SO_2_ when they are mixed with cirrus clouds. Moreover, another important limitation is related to the viewing angle, since the colors of a SEVIRI Ash RGB image depend on it. At high satellite viewing angles (>65°), it is difficult to correctly discriminate the volcanic cloud components, especially the SO_2_ gas because the water clouds appear on a green color similar to that of the SO_2_. When the satellite viewing angle is close to the sub-satellite point, the volcanic cloud components can be discriminated more easily and accurately. However, the main advantage of using this type of image is to easily recognize the different components of the volcanic cloud due to its intuitive colors [70]. These considerations led us to use the Ash RGB image as input feature of our SVM model. Thus, for each SEVIRI image, we selected the three TIR bands centered at 8.7, 10.8 and 12.0 µm and combined them to obtain an Ash RGB composite image.

Pixels of the Ash RGB image were normalized between 0 and 1 by applying Equation (1):

(1) $$x^{\prime }=\frac{x-x_{min}}{x_{max}-x_{min}}$$

where *x* is the pixel to normalize, $x_{min}$ the minimum pixel value of the image and $x_{max}$ the maximum pixel value of the image.

### 3.2. Classification

The SVM classifier was implemented in GEE to detect a volcanic cloud and to identify its main components, namely to classify its pixels as rich of ash, rich of SO_2_ or with mixed components (ash, SO_2_ and other). The SVM technique was already applied to volcano monitoring, and it was demonstrated that it provides successful results [71,72], due to its high performance and lower computation costs. Until now, algorithms and models have been developed for the retrieval of the components of a volcanic cloud [73,74,75] but without the implementation of artificial intelligence approaches. However, machine learning and deep learning techniques have been exploited mainly for the detection of a volcanic cloud [63,66,76,77].

We designed the SVM classifier using a radial basis function (RBF) as kernel, whose goal is to take data as input and transform them into the required form [78]. The RBF kernel function resembles Equation (2):

(2) $$k\left(x_{i},x_{j}\right)=exp(-\gamma {\left\|x_{i}-x_{j}\right\|}^{2})$$

where *γ* is the “spread” of the kernel and should be carefully tuned according to the problem. Another important parameter is the cost *c*, which is the penalty for misclassifying a data point. We set the parameter *γ* = 0.5 and the cost *c* = 10.

The realization of any supervised model such as SVM classifier consists of two parts, the training phase and the testing phase.

The first step in the training phase is to build a dataset of pixels from Ash RGB images labeled as pixel rich of ash belonging to a volcanic cloud (pure ash pixel), pixel rich of SO_2_ belonging to a volcanic cloud (pure SO_2_ pixel), pixel characterized by mixed components belonging to a volcanic cloud (mixed components pixel) and pixel not belonging to the cloud (background pixel). As a training dataset for the SVM classifier, small areas belonging to volcanic clouds are manually selected and are labeled as pure ash, pure SO_2_ or mixed components. Moreover, background regions are also defined and labeled. This approach is called multi-class classification, since the machine learning classification task consists of more than two classes [79,80]. In our case, the number of classes is four: (1) pure ash, (2) pure SO_2_, (3) mix of ash and SO_2_ and (4) background. The training samples are extracted from three SEVIRI Ash RGB images acquired on 23 February 2021 01:27 UTC, 23 February 2021 06:12 UTC and 12 March 2021 10:12 UTC. We chose these images because each of them presents a volcanic cloud with different composition, respectively, a pure-SO_2_ cloud, a pure-ash cloud and a cloud characterized by both ash and SO_2_. For each component, we used as training samples 10% of the total pixels related to that component contained in the three training images. For example, for the ash class, we used 10% of the total pure ash pixels contained in the three training images. Once the training dataset is built, the SVM model learns how to create the right output.

The second step is the testing phase; thus, the model learned by the training data is applied to the testing data, which are new SEVIRI Ash RGB images not containing training samples. To ensure that the trained model generalizes properly, it is important to verify the performance on different testing data that are not part of the training data.

We decided to use less training data than testing data to avoid the errors related to the labeling process, which is a manual process, and therefore to avoid ambiguity. In this way, we created a classifier as generalizable as possible that is able to discriminate the volcanic cloud from the background and to characterize its main components, resulting in an image with four classes (pure ash, pure SO_2_, mix and background).

In Table 1, the images used as training and as testing of the proposed SVM model are reported.

### 3.3. Performance Evaluation

Since we have a classification model for categorical classes, a confusion matrix can be used to evaluate the performance. The confusion matrix gives a comparison between actual and predicted values and is represented in a N × N matrix, where N is the number of classes. Each column of the matrix represents the instances in an actual class while each row represents the instances in a predicted class, or vice versa [81].

Let us consider the confusion matrix for a simple binary classification example. If we compare the actual classification values to the predicted classification values, there are 4 different outcomes:

- True positive (*TP*): the number of real positives that are correctly predicted as positive;
- False negative (*FN*): the number of real positives that are wrongly predicted as negative;
- False positive (*FP*): the number of real negatives that are wrongly predicted as positive;
- True negative (*TN*): the number of real negatives that are correctly predicted as negative.

The most common performance measures we can obtain from a confusion matrix are:

- *Precision*, which tells us what fraction of predicted positive values are actually positive

(3) $$precision=\frac{TP}{TP+FP};$$

- *Recall*, which tells us what fraction of all positive values are correctly predicted as positive by the classifier

(4) $$recall=\frac{TP}{TP+FN};$$

- *F*1*-score*, which combines precision and recall into a single measure

(5) $$F1-score=2\cdot \frac{precision\;\cdot \;recall}{precision+recall}=\frac{2\cdot TP}{2\cdot TP+FP+FN};$$

In the case of multi-class classification, we need to calculate *TP*, *FN*, *FP* and *TN* for each individual class [82]. Figure 2c shows the confusion matrix for a multi-class classification problem with four classes (Ash, SO_2_, Mix and Background). The dark green diagonal represents correct predictions, while the other light green cells indicate the incorrect predictions (*E*). As shown, *TP_ash_* is the number of true positive ash samples in class Ash, while *E_ash,SO*2*_* is the number of samples from class Ash that were incorrectly classified as class SO_2_. The false negative for the class Ash is the sum of *E_ash,SO*2*_*, *E_ash,mix_* and *E_ash,background_*, which indicates the sum of all class ash samples that were incorrectly classified as class SO_2_, Mix or Background. False positive for any predicted class, which is located in a row, represents the sum of all errors in that row.

The indices used to establish the reliability of our results are:

- Micro-averaged *F*1*-score* (*Micro-F*1**): it is calculated by using the regular *F*1*-score* formula considering the total TP, total *FP* and total *FN* of the model. It is a global metric since it does not consider each class individually.
- Macro-averaged *F*1*-score* (*Macro-F*1**): it is calculated by obtaining the metrics for each class individually, and then it takes the unweighted mean of the measures.
- Weighted-averaged *F*1*-score* (*Weighted-F*1**): it is calculated by taking a weighted mean of the measures.

In the case of a supervised machine learning model, we aim to build a model starting from a training dataset that also has very high performance indices in the test phase and therefore is able to work well with new dataset.

## 4. Results

The SVM classifier was trained on samples extracted from three SEVIRI Ash RGB images (see Table 1). First, the SVM model is applied to the SEVIRI Ash RGB images from which the training samples were extracted in order to classify the remaining pixels not used for the training. Each image is fed into the model, and for each case, we obtain as a result an outcome image in which all pixels are classified as pure ash, pure SO_2_, mix ash/SO_2_ or background.

**Figure 3:**
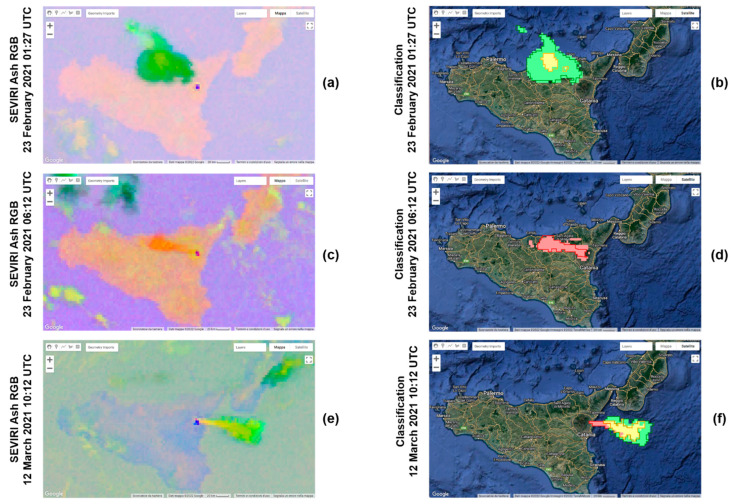
Input (SEVIRI Ash RGB images, left) and output (classification, right) of the SVM classifier for the 23 February 2021 01:27 UTC (**a**,**b**), 23 February 2021 06:12 UTC (**c**,**d**) and 12 March 2021 10:12 UTC (**e**,**f**) images, from GEE. These images are those from which the training sample were extracted.

In Figure 3a,c,e, we can see the SEVIRI Ash RGB images (23 February 2021 01:27 UTC, 23 February 2021 06:12 UTC and 12 March 2021 10:12 UTC) used as input of the SVM model, and in Figure 3b,d,f, the corresponding outcomes. These results are not used to evaluate the accuracy of the model, since training samples were extracted from these three images. For the testing phase, we selected three new images, where no training was performed on them. In Figure 3b,d,f, the pixels colored in red, green and yellow correspond, respectively, to the class pure-ash, pure SO_2_ and mixed components predicted by the SVM. The contours in red, green and yellow correspond, respectively, to the actual area characterized by pure ash, pure SO_2_ and mixed components, defined manually by visual inspection. Even visually, we can note that our algorithm classifies pretty well the pixels within a volcanic cloud.

**Figure 4:**
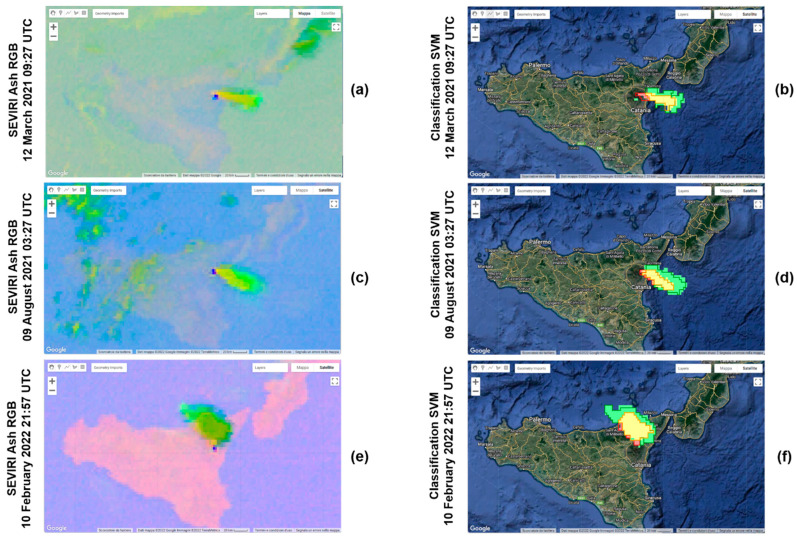
Input (SEVIRI Ash RGB images, left) and output (classification, right) of the SVM classifier for the 12 March 2021 09:27 UTC (**a**,**b**), 9 August 2021 03:27 UTC (**c**,**d**) and 10 February 2022 21:57 UTC (**e**,**f**) images, from GEE. No training was performed on these images.

In order to test the classifier, new images not used during the training phase are chosen. The SVM previously trained is applied to the SEVIRI Ash RGB images used as testing (see Table 1). The testing images were chosen in order to take into account volcanic clouds produced by eruptions from Etna that occurred in different and not consecutive periods in the 2020–2022 timeframe and therefore to make the evaluation of the accuracy of our algorithm more robust. In Figure 4a,c,e, we can see the SEVIRI Ash RGB images (12 March 2021 09:27 01:27 UTC, 9 August 2021 03:27 UTC and 10 February 2022 21:57 UTC) used as input of the SVM model, and in Figure 4b,d,f, the corresponding outcomes.

The accuracy of the proposed classifier was computed using the confusion matrix for the multi-class classification. For each testing image, we realized a confusion matrix, based on the areal extensions, and then, we extracted the respective indices (Micro-F1, Macro-F1 and Weighted-F1). Subsequently, we combined the confusion matrices of each testing images into one, summing the three matrices element by element, in order to obtain the 4 × 4 total confusion matrix. In Table 2, the confusion matrix with percentage values is shown. Starting from the total confusion matrix, we calculated the overall performance indices. In Table 3, the performance indices for the multi-class classification are collected, calculated considering the three testing images separately and finally using the total confusion matrix.

For the calculation of these performance indices, we did not take into account the values related to class background because the region outside the volcanic cloud is extremely larger than the one inside. Therefore, we would have a very great value of *TP_background_*, which would make the indices very high (also close to 1). Specifically, we are interested in evaluating the classifier’s ability to discriminate components within the cloud, for this reason, in the performance evaluation, we considered only the predicted and actual values related to ash, SO_2_ and mix.

## 5. Discussion

The SVM model has been found to be very efficient, since it is able to properly classify an image in a few minutes. Training samples were extracted from three images of reference, which are 23 February 2021 01:27 UTC, 23 February 2021 06:12 UTC and 12 March 2021 10:12 UTC. We decided to use these images because each of them contains a volcanic cloud which has different and specific characteristics. Specifically, the 23 February 2021 01:27 UTC image presents a pure-SO_2_ volcanic cloud, 23 February 2021 06:12 UTC image a pure-ash volcanic cloud, and 12 March 2021 10:12 UTC image, a cloud with both components.

First, we applied the SVM model to the three images from which the samples were extracted, and the results of the classification are shown in Figure 3b,d,f. We can see that the shape of the volcanic clouds is detected correctly, and most classified pixels belong to its real class. However, as the testing dataset, we used three new images where no training was performed, which are 12 March 2021 09:27 01:27 UTC, 9 August 2021 03:27 UTC and 10 February 2022 21:57 UTC. The results of the classification of the testing images are shown in Figure 4b,d,f.

To evaluate the performance of the algorithm, we took into account only the three images used as testing, and we created the confusion matrix reported in Table 2. On the vertical axis, we have the actual labels, while on the horizontal axis, the predicted labels of the testing samples. A perfect classifier is characterized by a confusion matrix with values only on the diagonal. If we look at the first column, which refers to the ash class, we can see that 76.8% of ash test samples is correctly classified, while the remaining 23.2% is wrongly predicted: 0.09% is classified as SO_2_, 6.5% as mix and 16.62% as background. This confusion matrix allowed us to retrieve the metrics for the measurements of the model, which are reported in Table 3. The performance indices *Micro-F*1**, *Macro-F*1** and *Weighted-F*1** are exploited to evaluate the ability of the model to correctly classify the volcanic cloud components. If we considered the three testing images separately, from Table 3, it is clear that the performances related to the first testing image (acquired during the day, at 09:27 UTC) are lower than the other two testing images (acquired during the night, respectively, at 03:27 and 21:57 UTC). Most probably, this is not related to the different times of day during which the acquisition took place, because SEVIRI Ash RGB images work well during both day and night periods, but to other factors. One of them is surely the greatest difficulty in recognizing the ash inside a cloud. This difficulty in discriminating the ash component is found especially when in a volcanic cloudthere are more components. In Figure 4b, we can see how part of the volcanic cloud containing ash was not classified as such, but as background. In contrast, in the case of a pure-ash volcanic cloud (such as that in Figure 3c), this component is detected very well (Figure 3d). On the whole, the values of these performance indices are always higher than 0.80 for the three testing images, and this means that the actual components present in a volcanic cloud are well predicted. Finally, we calculated the performance indices considering all the values of the four-class confusion matrix. In this way, we obtained an overall estimate of the indices *Micro-F*1**, *Macro-F*1** and *Weighted-F*1** (respectively, 0.86, 0.83 and 0.86), and we can conclude that the model performances are great.

We decided to use another machine learning algorithm to compare our SVM model. In particular, we implemented a random forest (RF) model, which is a classification algorithm consisting of many decisions trees. Each decision tree is trained independently using samples of the training dataset with replacement, and their results are combined to obtain the final RF outcome based on the majority vote [83]. This algorithm establishes the outcome based on the class selected by most trees. The RF is a supervised ML algorithm; therefore, we have used the same training dataset exploited to train our SVM model. The parameter to set in the RF is the number of trees. We performed four tests choosing as the number of trees 10, 50, 100 and 150. We observed that, in all four cases, the results obtained by this algorithm are acceptable, but in some cases, they are not very good.

**Figure 5:**
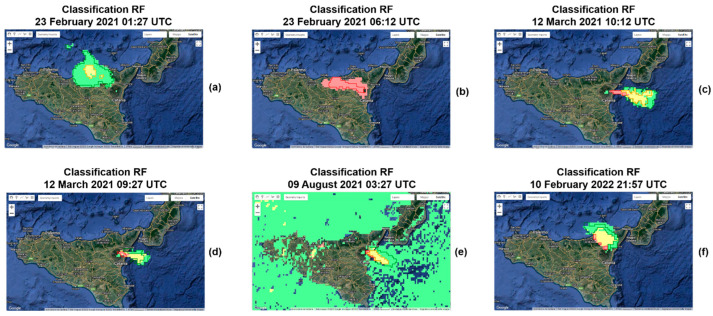
Output of the random forest (RF) classifier for the three images from which the training samples were extracted, (**a**) 23 February 2021 01:27 UTC, (**b**) 23 February 2021 06:12 UTC and (**c**) 12 March 2021 10:12 UTC and for the three testing images, (**d**) 12 March 2021 09:27 UTC, (**e**) 9 August 2021 03:27 UTC and (**f**) 10 February 2022 21:57 UTC images, from GEE.

In Figure 5, we reported the outcomes of the RF model, with a number of trees equal to 100, applied to the images of Table 1. The input of the model is a SEVIRI Ash RGB image. In Figure 5a–c, we can see the results of the classification of the three images from which the samples were extracted, whereas in Figure 5d–f, the results of the classification of the three testing images, where no training was performed, are shown.

By visual inspection, we can observe that the RF model detects volcanic clouds with an area greater than the real areal extension (Figure 5b,f). For the cases 23 February 2021 01:27 UTC, 12 March 2021 10:12 UTC and 12 March 2021 09:27 UTC, the RF model seems to work well (Figure 5a,c,d). However, in the case of 9 August 2021 03:27 UTC, the classification of RF does not work at all, because the SO_2_ is confused with most of the background (Figure 5e), while our SVM model is able to well discriminate the cloud from the background and classify its components (Figure 4d).

**Figure 6:**
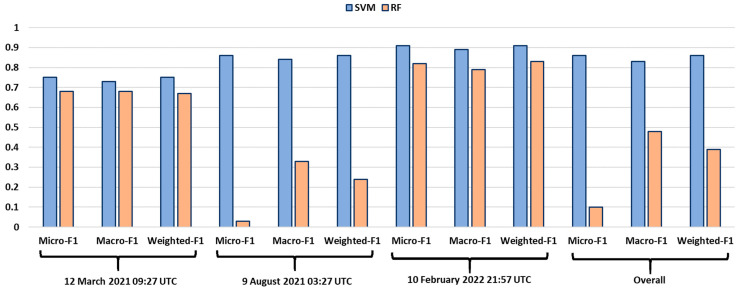
Comparison between the performance indices of SVM and RF.

The accuracy of the RF model is computed using the confusion matrix for the multi-class classification, and from this, we extracted the performance indices. In Figure 6, the comparison between the performance indices of the SVM and the RF is reported. The overall accuracy of the RF model results is very low, and this is mainly due to the fact that the image of 9 August 2021 03:27 UTC is not classified well.

We can conclude that our SVM model is more efficient than the RF for the task of detection and characterization of a volcanic cloud. It works well not only when it is applied to images from which the training samples are extracted (Figure 3a,c,e), but also with images where no training is performed (Figure 4a,c,e). Therefore, the possibility of applying the SVM model to new images makes it generalizable and applicable during a volcanic event in near real time to monitor the evolution of a volcanic cloud.

**Figure 7:**
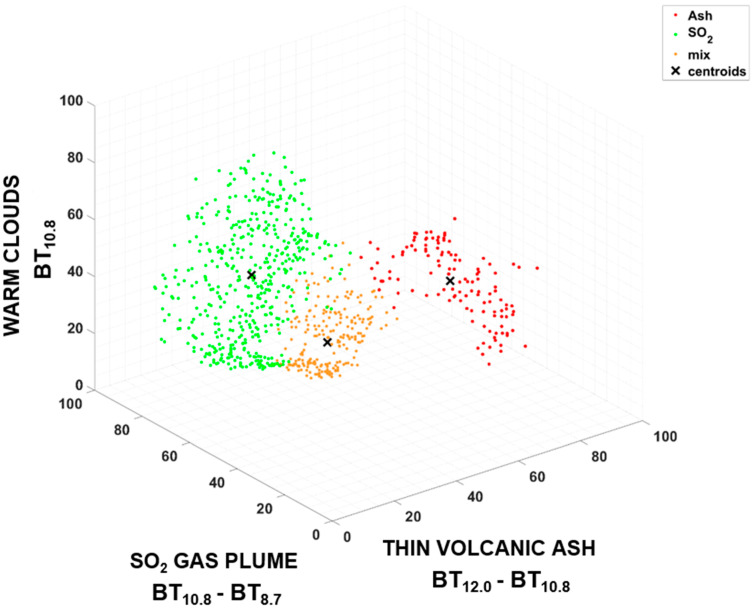
Three-dimensional plot with the normalized brightness temperature (BT) pixel values in a three-dimensional space where the axes are the channels of the SEVIRI Ash RGB images, and the colors of the points depend on their class group (red for ash, green for SO_2_ and yellow for mix). This plot shows the normalized BT values of the pixels belonging to the three images of reference (23 February 2021 01:27 UTC, 23 February 2021 06:12 UTC and 12 March 2021 10:12 UTC), without the training sample, and to the three testing images (12 March 2021 09:27 UTC, 9 August 2021 03:27 UTC and 10 February 2022 21:57 UTC).

In Figure 7, a 3D plot visualizing the BT values of the pixel in a three-dimensional space is shown, where the axes are the channels of the SEVIRI Ash RGB images, and each point has a different color according to its class group (red for ash, green for SO_2_, and yellow for mix of ash and SO_2_). The first channel (BT_12.0_ − BT_10.8_) emphasizes the presence of thin volcanic ash, the second (BT_10.8_ − BT_8.7_) the presence of SO_2_, and the third (BT_10.8_) the presence of warm clouds. Moreover, the pixel values of the three channels are normalized between 0 and 100. This plot shows the values of the pixels belonging to the three images of reference (23 February 2021 01:27 UTC, 23 February 2021 06:12 UTC and 12 March 2021 10:12 UTC), without the training sample, and to the three testing images (12 March 2021 09:27 UTC, 9 August 2021 03:27 UTC and 10 February 2022 21:57 UTC). For each of the three clusters, we established a centroid value calculated as the mean value of the points belonging to each class. The normalized brightness temperature values of the three centroids are reported in Table 4.

The ash centroid assumes a very high value in channel 1, which is the channel related to the presence of thin volcanic ash. Furthermore, the SO_2_ centroid has a high value in channel 2, which is related to the presence of SO_2_ gas plume. Lastly, the mix centroid has high value both for channel 1 and channel 2, since the pixels classified as mix contain both ash and SO_2_. Then, the pixels classified as ash will be on the right side of the 3D graph, which is the part with the highest values in channel 1. The pixels classified as SO_2_ will instead be concentrated on the left side of the 3D graph, where there are the highest values of channel 2. Instead, the pixels classified as mix will be in a position between the ash class and the SO_2_ class. A SEVIRI pixel has a rough spatial resolution (around 16 km^2^ at the considered latitudes); thus, being very large, it can contain more components that can be difficult to discriminate. For this reason, it can happen that some pixels may not be correctly classified and will be far from the centroid to which they have been assigned. Furthermore, misclassification may be due to the dependence of the colors on the satellite viewing angle and the difficult identification of ash and SO_2_ when they are mixed with cirrus clouds. However, we found that the pixels of each class are well distributed around their own centroid. Since we have verified that the accuracy of our model is high, we can say that the centroids have been correctly determined.

**Figure 8:**
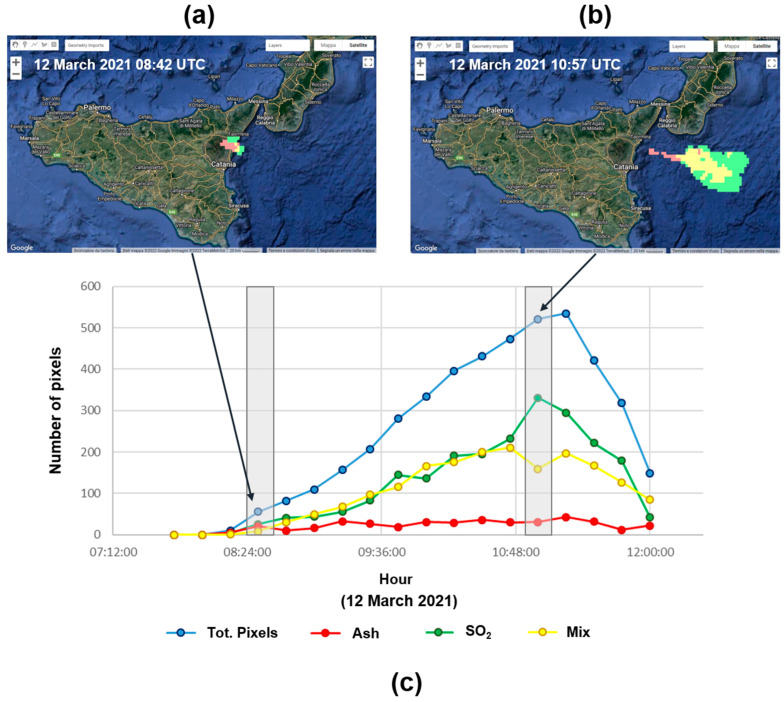
SVM outcomes for the 12 March 2021 08:42 UTC (**a**) and the 12 March 2021 10:57 UTC (**b**) images and tracking of major components of the volcanic cloud produced during the 12 March 2021 explosive event at Etna volcano (**c**).

The proposed SVM model and the high temporal resolution of SEVIRI give us the possibility to visualize and follow a volcanic cloud during an eruptive episode, from its formation to its complete dispersion in the atmosphere. In Figure 8, the tracking of the major components of the volcanic cloud produced during the 12 March 2021 event at Etna volcano is represented. This analysis was conducted considering 18 SEVIRI Ash RGB images acquired on 12 March 2021 (from 07:42 UTC to 11:57 UTC). The SVM model was applied to each image. The graph in Figure 8 shows the total number of pixels inside the detected volcanic cloud and the number of pixels related to each component inside the cloud. The volcanic cloud is visible from the 08:12 UTC, and its dimensions increase until 11:12 UTC. Afterward, the volcanic cloud starts to disperse into the atmosphere, and the number of pixels begins to decrease progressively. Thus, we demonstrated how our SVM model combined with the high sample rate of SEVIRI can be exploited to track the dispersion of a volcanic cloud in near real time.

## 6. Conclusions

Accurate detection, tracking, and ultimately nowcasting (i.e., near real time tracking and short-term forecasting) of volcanic ash clouds combining high temporal resolution satellite data and machine learning techniques have immediate applications to the real-time monitoring of volcanic explosive eruptions. By monitoring, we mean here both following the manifestations of the eruption once it has started as well as forecasting the areas potentially threatened by major components of a volcanic cloud. The need for integrated and efficient monitoring systems, operating on a global scale, and including tools for producing different scenarios as eruptive conditions change, is a primary challenge for volcanic hazard assessment.

We described and demonstrated the operation of a SVM classifier designed for the detection of volcanic clouds and the classification of their main components. The results show a unique temporal dataset of major volcanic cloud components, from their formation to their dispersion in the atmosphere, hosted and processed by a cloud computing platform. This enabled the rapid assessment of eruption evolution via a cloud computing platform that can collect and process time series data within minutes.

We validated our approach in an operational context at Etna volcano during the paroxysmal explosive events that occurred between 2020 and 2022. As input of the classifier, we used Ash RGB composite images generated using a combination of three TIR bands from SEVIRI. The advantage of this model is that, once trained, due to its good accuracy, it can be applied to new images without having to be trained again. As a result, the proposed SVM is able to detect and characterize the volcanic cloud in any new image, thus allowing one to detect and track the entire volcanic event due to SEVIRI high temporal resolution. This approach gives us the possibility to monitor the evolution of a volcanic cloud in near real time and therefore to understand which regions may be most affected by its impact, since the emissions of ash and SO_2_ can be hazardous to public health.

Although further analyses are required to fully evaluate the performance of the SVM model, it may support operational monitoring centers, such as the Etna Volcano Observatory (EVO), involved in better managing of volcanic ash clouds, providing time-critical hazard information. Therefore, the developed technology is expected to improve operational hazard detection, alerting, and management capabilities, minimizing the impact of dangerous and highly destructive paroxysmal events on populations and the environment.

## Figures and Tables

**Table 1:** List of images used as training and as testing of the proposed SVM model.

Training Images		Testing Images	
23 February 2021 01:27 UTC	MSG4-SEVI-MSG15-0100-NA-20210223012743.577000000Z-NA	12 March 2021 09:27 UTC	MSG4-SEVI-MSG15-0100-NA-20210312092743.251000000Z-NA
23 February 2021 06:12 UTC	MSG4-SEVI-MSG15-0100-NA-20210223061243.447000000Z-NA	9 August 2021 03:27 UTC	MSG4-SEVI-MSG15-0100-NA-20210809032743.000000000Z-NA
12 March 2021 10:12 UTC	MSG4-SEVI-MSG15-0100-NA-20210312101243.794000000Z-NA	10 February 2022 21:57 UTC	MSG4-SEVI-MSG15-0100-NA-20220210215742.231000000Z-NA

**Table 2:** Overall confusion matrix with percentage values.

		Actual Values			
		Ash	SO_2_	Mix	Background
**Predicted Values**	**Ash**	76.8%	0.01%	3.21%	0.01%
	**SO_2_**	0.09%	77.9%	1.84%	0.09%
	**Mix**	6.5%	5.64%	93.74%	0.01%
	**Background**	16.62%	16.45%	1.22%	99.89%

**Table 3:** Performance indices for multi-class classification.

SEVIRI Image	Micro-F1	Macro-F1	Weighted-F1
12 March 2021 09:27 UTC	0.75	0.73	0.75
9 August 2021 03:27 UTC	0.86	0.84	0.86
10 February 2022 21:57 UTC	0.91	0.89	0.91
Overall	0.86	0.83	0.86

**Table 4:** Normalized brightness temperature values of centroid for ash, SO_2_ and mix classes.

Centroids	Channel 1	Channel 2	Channel 3
	THIN VOLCANIC ASH	SO_2_ GAS PLUME	WARM CLOUDS
Ash	86.74	63.49	24.68
SO_2_	34.51	78.94	38.01
Mix	57.48	76.95	7.19

## Data Availability

Data used in this paper can be downloaded from EUMETSAT’s website.

## References

[1] Durant A.J., Bonadonna C., Horwell C.J. (2010). Atmospheric and Environmental Impacts of Volcanic Particulates. Elements.

[2] Thomas H.E., Prata A.J. (2011). Sulphur Dioxide as a Volcanic Ash Proxy during the April–May 2010 Eruption of Eyjafjallajökull Volcano, Iceland. Atmos. Chem. Phys..

[3] Thordarson T., Self S. (2003). Atmosphere and Environmental Effects of the 1783-1784 Laki Eruption: A Review and Reassessment. J. Geophys. Res..

[4] Colette A., Favez O., Meleux F., Chiappini L., Haeffelin M., Morille Y., Malherbe L., Papin A., Bessagnet B., Menut L. (2011). Assessing in near Real Time the Impact of the April 2010 Eyjafjallajökull Ash Plume on Air Quality. Atmos. Environ..

[5] Casadevall T.J. (1994). The 1989–1990 Eruption of Redoubt Volcano, Alaska: Impacts on Aircraft Operations. J. Volcanol. Geotherm. Res..

[6] Horwell C.J., Baxter P.J. (2006). The Respiratory Health Hazards of Volcanic Ash: A Review for Volcanic Risk Mitigation. Bull. Volcanol..

[7] Osiensky J., Hall T. (2008). Detection and Tracking of Volcanic Ash and SO2 and Its Impact to Aviation. AGU Fall Meeting Abstract.

[8] McCormick M.P., Thomason L.W., Trepte C.R. (1995). Atmospheric Effects of the Mt Pinatubo Eruption. Nature.

[9] Robock A. (2000). Volcanic Eruptions and Climate. Rev. Geophys..

[10] Gislason S.R., Hassenkam T., Nedel S., Bovet N., Eiriksdottir E.S., Alfredsson H.A., Hem C.P., Balogh Z.I., Dideriksen K., Oskarsson N. (2011). Characterization of Eyjafjallajökull Volcanic Ash Particles and a Protocol for Rapid Risk Assessment. Proc. Natl. Acad. Sci. USA.

[11] Jenkins S.F., Wilson T.M., Magill C.R., Miller V., Stewart C., Marzocchi W., Boulton M. (2014). Volcanic Ash Fall Hazard and Risk: Technical Background Paper for the UN-ISDR 2015 Global Assessment Report on Disaster Risk Reduction.

[12] Loughlin S.C., Sparks R.S.J., Sparks S., Brown S.K., Jenkins S.F., Vye-Brown C. (2015). Global Volcanic Hazards and Risk.

[13] U.S. Government (1994). Volcanic Ash and Aviation Safety: Proceedings of the First International Symposium on Volcanic Ash and Aviation Safety.

[14] National Geographic Society Human and Environmental Impacts of Volcanic Ash. https://education.nationalgeographic.org/resource/human-environmental-impact-volcanic-ash.

[15] The Health Hazards of Volcanoes and Geothermal Areas Occupational & Environmental Medicine. https://oem.bmj.com/content/63/2/149.short.

[16] Rose W.I., Bluth G.J.S., Ernst G.G.J. (2000). Integrating Retrievals of Volcanic Cloud Characteristics from Satellite Remote Sensors: A Summary. Philos. Trans. Math. Phys. Eng. Sci..

[17] Watson I.M., Realmuto V.J., Rose W.I., Prata A.J., Bluth G.J.S., Gu Y., Bader C.E., Yu T. (2004). Thermal Infrared Remote Sensing of Volcanic Emissions Using the Moderate Resolution Imaging Spectroradiometer. J. Volcanol. Geotherm. Res..

[18] Corradini S., Montopoli M., Guerrieri L., Ricci M., Scollo S., Merucci L., Marzano F., Pugnaghi S., Prestifilippo M., Ventress L. (2016). A Multi-Sensor Approach for Volcanic Ash Cloud Retrieval and Eruption Characterization: The 23 November 2013 Etna Lava Fountain. Remote Sens..

[19] Dubuisson P., Herbin H., Minvielle F., Compiègne M., Thieuleux F., Parol F., Pelon J. (2014). Remote Sensing of Volcanic Ash Plumes from Thermal Infrared: A Case Study Analysis from SEVIRI, MODIS and IASI Instruments. Atmos. Meas. Tech..

[20] Prata F., Lynch M. (2019). Passive Earth Observations of Volcanic Clouds in the Atmosphere. Atmosphere.

[21] Fensholt R., Anyamba A., Huber S., Proud S.R., Tucker C.J., Small J., Pak E., Rasmussen M.O., Sandholt I., Shisanya C. (2011). Analysing the Advantages of High Temporal Resolution Geostationary MSG SEVIRI Data Compared to Polar Operational Environmental Satellite Data for Land Surface Monitoring in Africa. Int. J. Appl. Earth Obs. Geoinf..

[22] Sawada Y. (1987). Study on Analyses of Volcanic Eruptions Based on Eruption Cloud Image Data Obtained by the Geostationary Meteorological Satellite(GMS). Tech. Rep. Meteorol. Res. Inst..

[23] Prata A.J. (1989). Infrared Radiative Transfer Calculations for Volcanic Ash Clouds. Geophys. Res. Lett..

[24] Prata F. (1989). Observations of Volcanic Ash Clouds in the 10–12 μm Window Using AVHRR/2 Data. Int. J. Remote Sens..

[25] Prata A.T. (2017). Active and Passive Satellite Remote Sensing of Volcanic Clouds. Ph.D. Thesis.

[26] Yu T., Rose W.I., Prata A.J. (2002). Atmospheric Correction for Satellite-Based Volcanic Ash Mapping and Retrievals Using “Split Window” IR Data from GOES and AVHRR. J. Geophys. Res. Atmos..

[27] Simpson J., Hufford G., Pieri D., Berg J. (2000). Failures in Detecting Volcanic Ash from a Satellite-Based Technique. Remote Sens. Environ..

[28] Comments on “Failures in Detecting Volcanic Ash from a Satellite-Based Technique”—ScienceDirect. https://www.sciencedirect.com/science/article/pii/S0034425701002310.

[29] Ellrod G.P., Connell B.H., Hillger D.W. (2003). Improved Detection of Airborne Volcanic Ash Using Multispectral Infrared Satellite Data. J. Geophys. Res. Atmos..

[30] Pergola N., Valerio T., Marchese F., Scaffidi I., Lacava T. (2004). Improving Volcanic Ash Cloud Detection by a Robust Satellite Technique. Remote Sens. Environ..

[31] Ellrod G.P., Schreiner A.J. (2004). Volcanic Ash Detection and Cloud Top Height Estimates from the GOES-12 Imager: Coping without a 12 Μm Infrared Band. Geophys. Res. Lett..

[32] Francis P.N., Cooke M.C., Saunders R.W. (2012). Retrieval of Physical Properties of Volcanic Ash Using Meteosat: A Case Study from the 2010 Eyjafjallajökull Eruption. J. Geophys. Res. Atmos..

[33] Pavolonis M.J., Heidinger A.K., Sieglaff J. (2013). Automated Retrievals of Volcanic Ash and Dust Cloud Properties from Upwelling Infrared Measurements. J. Geophys. Res. Atmos..

[34] Prata A.J., Kerkmann J. (2007). Simultaneous Retrieval of Volcanic Ash and SO_2_ Using MSG-SEVIRI Measurements. Geophys. Res. Lett..

[35] Corradini S., Merucci L., Prata A.J., Piscini A. (2010). Volcanic Ash and SO_2_ in the 2008 Kasatochi Eruption: Retrievals Comparison from Different IR Satellite Sensors. J. Geophys. Res. Atmos..

[36] Wen S., Rose W.I. (1994). Retrieval of Sizes and Total Masses of Particles in Volcanic Clouds Using AVHRR Bands 4 and 5. J. Geophys. Res. Atmos..

[37] Realmuto V.J., Berk A. (2016). Plume Tracker: Interactive Mapping of Volcanic Sulfur Dioxide Emissions with High-Performance Radiative Transfer Modeling. J. Volcanol. Geotherm. Res..

[38] Berk A., Bernstein L., Robertson D. (1987). MODTRAN: A Moderate Resolution Model for LOWTRAN.

[39] Corradini S., Guerrieri L., Stelitano D., Salerno G., Scollo S., Merucci L., Prestifilippo M., Musacchio M., Silvestri M., Lombardo V. (2020). Near Real-Time Monitoring of the Christmas 2018 Etna Eruption Using SEVIRI and Products Validation. Remote Sens..

[40] Corradini S., Merucci L., Prata A.J. (2009). Retrieval of SO_2_ from Thermal Infrared Satellite Measurements: Correction Procedures for the Effects of Volcanic Ash. Atmos. Meas. Tech..

[41] Prata A.J., Grant I.F. (2001). Determination of Mass Loadings and Plume Heights of Volcanic Ash Clouds from Satellite Data.

[42] Corradini S., Spinetti C., Carboni E., Tirelli C., Buongiorno M.F., Pugnaghi S., Gangale G. (2008). Mt. Etna Tropospheric Ash Retrieval and Sensitivity Analysis Using Moderate Resolution Imaging Spectroradiometer Measurements. J. Appl. Remote Sens..

[43] Corradino C., Ganci G., Cappello A., Bilotta G., Hérault A., Del Negro C. (2019). Mapping Recent Lava Flows at Mount Etna Using Multispectral Sentinel-2 Images and Machine Learning Techniques. Remote Sens..

[44] Corradino C., Amato E., Torrisi F., Calvari S., Del Negro C. (2021). Classifying Major Explosions and Paroxysms at Stromboli Volcano (Italy) from Space. Remote Sens..

[45] Amato E., Corradino C., Torrisi F., Del Negro C. (2021). Combined Use of Satellite Data and Machine Learning for Detecting, Measuring, and Monitoring Active Lava Flows at Etna Volcano. Earth Space Sci. Open Arch..

[46] Del Negro C., Amato E., Torrisi F., Corradino C., Bucolo M., Fortuna L. Support Vector Machine for Volcano Hazard Monitoring from Space at Mount Etna. Proceedings of the IEEE MELECON.

[47] (2022). Torrisi, Federica Automatic Detection of Volcanic Ash Clouds Using MSG-SEVIRI Satellite Data and Machine Learning Techniques. Il Nuovo Cim. C.

[48] Gorelick N., Hancher M., Dixon M., Ilyushchenko S., Thau D., Moore R. (2017). Google Earth Engine: Planetary-Scale Geospatial Analysis for Everyone. Remote Sens. Environ..

[49] Bisson M., Spinetti C., Andronico D., Palaseanu-Lovejoy M., Fabrizia Buongiorno M., Alexandrov O., Cecere T. (2021). Ten Years of Volcanic Activity at Mt Etna: High-Resolution Mapping and Accurate Quantification of the Morphological Changes by Pleiades and Lidar Data. Int. J. Appl. Earth Obs. Geoinf..

[50] Behncke B., Branca S., Corsaro R.A., De Beni E., Miraglia L., Proietti C. (2014). The 2011–2012 Summit Activity of Mount Etna: Birth, Growth and Products of the New SE Crater. J. Volcanol. Geotherm. Res..

[51] Andronico D., Di Roberto A., De Beni E., Behncke B., Bertagnini A., Del Carlo P., Pompilio M. (2018). Pyroclastic Density Currents at Etna Volcano, Italy: The 11 February 2014 Case Study. J. Volcanol. Geotherm. Res..

[52] Andronico D., Behncke B., De Beni E., Cristaldi A., Scollo S., Lopez M., Lo Castro M.D. (2018). Magma Budget From Lava and Tephra Volumes Erupted During the 25-26 October 2013 Lava Fountain at Mt Etna. Front. Earth Sci..

[53] Calvari S., Bonaccorso A., Ganci G. (2021). Anatomy of a Paroxysmal Lava Fountain at Etna Volcano: The Case of the 12 March 2021, Episode. Remote Sens..

[54] Marchese F., Filizzola C., Lacava T., Falconieri A., Faruolo M., Genzano N., Mazzeo G., Pietrapertosa C., Pergola N., Tramutoli V. (2021). Mt. Etna Paroxysms of February–April 2021 Monitored and Quantified through a Multi-Platform Satellite Observing System. Remote Sens..

[55] Calvari S., Nunnari G. (2022). Comparison between Automated and Manual Detection of Lava Fountains from Fixed Monitoring Thermal Cameras at Etna Volcano, Italy. Remote Sens..

[56] (2022). Amato, Eleonora Machine Learning and Best Fit Approach to Map Lava Flows from Space. Il Nuovo Cim. C.

[57] Aminou D.M.A., Jacquet B., Pasternak F. (1997). Characteristics of the Meteosat Second Generation (MSG) Radiometer/Imager: SEVIRI. Proc. Sens. Syst. Next Gener. Satell..

[58] Aminou D. (2002). MSG’s SEVIRI Instrument. ESA Bull..

[59] Sain S.R. (1996). The Nature of Statistical Learning Theory. Technometrics.

[60] Xu Y., Zomer S., Brereton R.G. (2006). Support Vector Machines: A Recent Method for Classification in Chemometrics. Crit. Rev. Anal. Chem..

[61] Ben-Hur A., Weston J., Carugo O., Eisenhaber F. (2010). A User’s Guide to Support Vector Machines. Data Mining Techniques for the Life Sciences.

[62] Gardner M.W., Dorling S.R. (1998). Artificial Neural Networks (the Multilayer Perceptron)—A Review of Applications in the Atmospheric Sciences. Atmos. Environ..

[63] Picchiani M., Chini M., Corradini S., Merucci L., Sellitto P., Del Frate F., Stramondo S. (2011). Volcanic Ash Detection and Retrievals Using MODIS Data by Means of Neural Networks. Atmos. Meas. Tech..

[64] Sellitto P., Del Frate F., Solimini D., Casadio S. (2012). Tropospheric Ozone Column Retrieval From ESA-Envisat SCIAMACHY Nadir UV/VIS Radiance Measurements by Means of a Neural Network Algorithm. IEEE Trans. Geosci. Remote Sens..

[65] Di Noia A., Sellitto P., Del Frate F., de Laat J. (2013). Global Tropospheric Ozone Column Retrievals from OMI Data by Means of Neural Networks. Atmos. Meas. Tech..

[66] Picchiani M., Chini M., Corradini S., Merucci L., Piscini A., Frate F.D. (2014). Neural Network Multispectral Satellite Images Classification of Volcanic Ash Plumes in a Cloudy Scenario. Ann. Geophys..

[67] Piscini A., Picchiani M., Chini M., Corradini S., Merucci L., Del Frate F., Stramondo S. (2014). A Neural Network Approach for the Simultaneous Retrieval of Volcanic Ash Parameters and SO_2_ Using MODIS Data. Atmos. Meas. Tech..

[68] EUMETSAT MSG Level 1.5 Image Data Format Description. https://www-cdn.eumetsat.int/files/2020-05/pdf_ten_05105_msg_img_data.pdf.

[69] SEVIRI Ash RGB Quick Guide. www-cdn.eumetsat.int/files/2020-04/pdf_rgb_quick_guide_ash.pdf.

[70] Torrisi F., Folzani F., Corradino C., Amato E., Negro C.D. (2022). Detecting Volcanic Ash Plume Components from Space Using Machine Learning Techniques. Earth Space Sci. Open Arch..

[71] Corradino C., Bilotta G., Cappello A., Fortuna L., Del Negro C. (2021). Combining Radar and Optical Satellite Imagery with Machine Learning to Map Lava Flows at Mount Etna and Fogo Island. Energies.

[72] Amato E., Corradino C., Torrisi F., Del Negro C. Mapping Lava Flows at Etna Volcano Using Google Earth Engine, Open-Access Satellite Data, and Machine Learning. Proceedings of the 2021 International Conference on Electrical, Computer, Communications and Mechatronics Engineering (ICECCME).

[73] Guerrieri L., Merucci L., Corradini S., Pugnaghi S. (2015). Evolution of the 2011 Mt. Etna Ash and SO2 Lava Fountain Episodes Using SEVIRI Data and VPR Retrieval Approach. J. Volcanol. Geotherm. Res..

[74] Pugnaghi S., Guerrieri L., Corradini S., Merucci L., Arvani B. (2013). A New Simplified Approach for Simultaneous Retrieval of SO_2_ and Ash Content of Tropospheric Volcanic Clouds: An Application to the Mt Etna Volcano. Atmos. Meas. Tech..

[75] Pugnaghi S., Guerrieri L., Corradini S., Merucci L. (2016). Real Time Retrieval of Volcanic Cloud Particles and SO_2_ by Satellite Using an Improved Simplified Approach. Atmos. Meas. Tech..

[76] Gray T.M., Bennartz R. (2015). Automatic Volcanic Ash Detection from MODIS Observations Using a Back-Propagation Neural Network. Atmos. Meas. Tech..

[77] Piontek D., Bugliaro L., Kar J., Schumann U., Marenco F., Plu M., Voigt C. (2021). The New Volcanic Ash Satellite Retrieval VACOS Using MSG/SEVIRI and Artificial Neural Networks: 2. Validation. Remote Sens..

[78] Corradino C., Amato E., Torrisi F., Negro C.D. Towards an Automatic Generalized Machine Learning Approach to Map Lava Flows. Proceedings of the 2021 17th International Workshop on Cellular Nanoscale Networks and their Applications (CNNA).

[79] Mayoraz E., Alpaydin E., Mira J., Sánchez-Andrés J.V. (1999). Support Vector Machines for Multi-Class Classification. Proceedings of the Engineering Applications of Bio-Inspired Artificial Neural Networks.

[80] Grandini M., Bagli E., Visani G. (2020). Metrics for Multi-Class Classification: An Overview. arXiv.

[81] Powers D.M.W. (2020). Evaluation: From Precision, Recall and F-Measure to ROC, Informedness, Markedness and Correlation. arXiv.

[82] Tharwat A. (2020). Classification Assessment Methods. Appl. Comput. Inform..

[83] Corradino C., Amato E., Torrisi F., Del Negro C. (2022). Data-Driven Random Forest Models for Detecting Volcanic Hot Spots in Sentinel-2 MSI Images. Remote Sens..

